# Intravenous injection of neural progenitor cells facilitates angiogenesis after cerebral ischemia

**DOI:** 10.1002/brb3.113

**Published:** 2012-12-28

**Authors:** Yoshiyuki Moriyama, Norio Takagi, Kanae Hashimura, Chisa Itokawa, Kouichi Tanonaka

**Affiliations:** Department of Molecular and Cellular Pharmacology, Tokyo University of Pharmacy and Life Sciences1432-1 Horinouchi, Hachioji, Tokyo, 192-0392, Japan

**Keywords:** Angiopoietin, cerebral ischemia, neural progenitor cells, tight junctional protein, VEGF

## Abstract

Earlier we demonstrated that the injection of neural progenitor cells (NPCs) has therapeutic potential for the improvement of learning dysfunction after cerebral ischemia. However, it remained to be clarified how transplantation of NPCs can improve ischemia-induced dysfunction. In this study, we examined whether intravenous injection of NPCs after cerebral ischemia could enhance angiogenesis by affecting the expression of angiogenic factors. The injection of NPCs on day 7 after cerebral ischemia enhanced angiogenesis on day 28. Vascular endothelial growth factor (VEGF) and its receptor VEGFR2 were increased in expression by the NPC injection. The level of angiopoietin-1 (Ang-1), an angiogenic factor, but not that of Ang-2, which acts as an antagonist for the Ang-1 receptor, was also increased on day 28. In addition, the expression of Ang-1 receptor Tie2 was enhanced in brain capillaries. Furthermore, the amounts of tight junctional proteins, which are in the blood–brain barrier and whose expression occurs downstream of Ang-1/Tie2 signaling, were increased by the NPC injection. These results suggest that the NPC injection promoted angiogenesis through Ang-1/Tie2 and/or VEGF/VEGFR2 signaling in brain capillaries after cerebral ischemia. Such signaling might have the potential for causing vascular stabilization and maturation for a long period after cerebral ischemia.

## Introduction

Stroke is the leading cause of disability and mortality (Lloyd-Jones et al. 2009). Despite a wealth of insight into the pathogenesis of stroke, current therapies for this devastating disease are far from optimal. Beyond the narrow therapeutic time window open for thrombolysis, only rehabilitation appears to be effective. Therefore, stroke survivors suffer severe aftereffects, including motor dysfunction, cognitive impairment, and mood disorder.

In general, the supply of oxygen and nutritional factors from bloodstream is required for cell differentiation and maturation. To be supplied with oxygen and nutritional factor in ischemic tissues, angiogenic activity would be required. It has been suggested that angiogenesis and vascular maturation are regulated by vascular endothelial growth factor (VEGF) and angiopoietin-1 (Ang-1). VEGF receptor 2 (Flk1) and Ang-1 receptor Tie2 have tyrosine kinase domains that play essential roles in angiogenesis (Patan 2004; Pfaff et al. 2006). VEGF is upregulated in the central nervous system (CNS) after injury (Hayashi et al. 1997; Skold et al. 2005; Wang et al. 2005; Dore-Duffy et al. 2007) and induces mature and/or immature angiogenesis (Nag et al. 1997; Nieto et al. 2001). VEGF also has beneficial effects on the survival of newborn neuronal precursors (Widenfalk et al. 2003) and has been implicated in neurogenesis after cerebral ischemia (Jin et al. 2002; Sun et al. 2003; Wang et al. 2007) and in neurite outgrowth (Jin et al. 2006). Vascular maturation and stabilization are required for functional angiogenesis (Hirschi et al. 1997). In this sense, Ang-1 reduces endothelial permeability and enhances vascular stabilization and maturation (Suri et al. 1998). Furthermore, Ang-1 signaling promotes angiogenesis and remodeling of blood vessels through its receptor tyrosine kinase Tie2, which is expressed on endothelial cells of blood vessel.

Cell-based therapy, including the use of neural stem and progenitor cells or bone marrow stromal cells, has shown potential to regenerate and repair the CNS in several types of animal models for brain ischemia (Zhao et al. 2002; Taguchi et al. 2004; Kozlowska et al. 2007; Mochizuki et al. 2008; Takahashi et al. 2008). However, it remains to be clarified how transplantation of NPCs can improve ischemia-induced dysfunction. Microsphere embolism model used in this study has been shown to induce widespread formation of small permanent emboli in the ipsilateral hemisphere and severe spatial learning and memory dysfunction (Miyake et al. 1993; Nagakura et al. 2002; Date et al. 2004). Therefore, microsphere embolism model is considered to mimic focal ischemia-induced human stroke and/or multi-infarct dementia (Naritomi 1991; Lyden et al. 1992). In previous studies, we isolated NPCs and injected them intravenously on day 7 after the induction of cerebral embolism to avoid the inappropriate environment for therapeutic injection of NPCs that would exist immediately after a stroke (Mochizuki et al. 2011; Moriyama et al. 2011). These studies demonstrated that the intravenous injection of NPCs improves motor function, spatial learning dysfunction, and depression-like behavior after cerebral ischemia (Mochizuki et al. 2011; Moriyama et al. 2011). However, it has not been reported whether intravenous administration of NPC at a relatively late stage after cerebral embolic model, which induces severe learning and memory dysfunction and poststroke depression-like behavior, can affect the level of angiogenic factors. The changes in angiogenesis at a longer period of time after the induction of ischemia may be associated with the improvement of learning dysfunction and depression-like behaviors. Therefore, in this study, we investigated whether the intravenous injection of NPCs on day 7 after a cerebral embolism would facilitate angiogenesis. We did so by examining the expression of VEGF/Flk1 and Ang1/Tie2, either or both of which might be expected to promote angiogenesis.

## Materials and Methods

### Model of microsphere-induced cerebral embolism in rats

Male Wistar rats weighing 220–250 g (Charles River Japan, Inc., Tsukuba, Japan) were used. The rats were maintained at 23 ± 1°C in a room with a constant humidity of 55 ± 5% and a light cycle of 12-h light:12-h darkness. The rats had free access to food and water according to the National Institute of Health Guide for the Care and Use of Laboratory Animals and the Guidance for Experimental Animal Care issued by the Prime Minister's Office of Japan. The study was approved by the Committee of Animal Care and Welfare of Tokyo University of Pharmacy and Life Sciences. Microsphere-induced cerebral embolism (ME) was performed by the method described previously (Mochizuki et al. 2008). After rats had been anesthetized by 40 mg/kg sodium pentobarbital, the right external carotid and pterygopalatine arteries were temporarily occluded with strings. Immediately, a needle connected to a polyethylene catheter (TORAY Feeding Tube, Chiba, Japan) was inserted into the right common carotid artery, and then 700 microspheres (45.0 μm in diameter; Polysciences, Inc., Warrington, PA), suspended in 20% dextran solution, were injected into the right internal carotid artery through the cannula. After the injection, the needle was removed, and the puncture wound was repaired with surgical glue. The rats that underwent a sham operation received the same volume of vehicle without microspheres.

### NPC cultures

NPCs were prepared from gestational day 14 fetal Wistar rats, as described previously (Mochizuki et al. 2008; Moriyama et al. 2011). Cells were seeded at a density of 50,000 cells/cm^2^ into nontreated flasks (Nalge Nunc International, New York, NY) containing N-2 plus medium supplemented with 20 ng/mL epidermal growth factor and 20 ng/mL basic fibroblast growth factor (b-FGF; growth medium). The NPCs were grown in culture as free-floating neurospheres, and 80% of the medium was exchanged for new growth medium on day 4. For the experiments, neurospheres cultured for 6 days in vitro were used. In some experiments, NPCs were prepared from gestational day-14 fetal green-fluorescent protein (GFP) transgenic rats, as described previously (Moriyama et al. 2011). The GFP transgenic rats (Wistar-TgN [CAG-GFP] 184ys) used in this study were provided by National Bio Resource Project for the Rat in Japan (Kyoto, Japan). The origin and characteristics of the transgenic rats were described previously (Hakamata et al. 2001). The protocol was approved by the Committee of Animal Care and Welfare of Tokyo University of Pharmacy and Life Sciences.

For immunostaining of floating cultured neurospheres, they were attached by incubation on poly-l-lysine (Sigma-Aldrich, St. Louis, MO)-coated slides for 1 h at 25°C and then fixed for 30 min with 4% paraformaldehyde. The primary antibodies used for neurospheres were rat monoclonal anti-GFP (Nacalai Tesque, Kyoto, Japan) and rabbit polyclonal anti-musashi-1 (Chemicon, Temecula, CA). Omission of primary antibodies served as negative controls. No immunostaining was detected in the group of negative controls.

For differentiation, neurospheres were cultured for 6 days in vitro followed by replacement of the medium with Dulbecco's modified Eagle medium (DMEM)/F12 medium without EGF and b-FGF on day 7. The neurospheres were then cultured for seven additional days. For immunostaining of differentiated NPCs, the following primary antibodies were used: mouse monoclonal anti-MAP2 (Sigma-Aldrich, St. Louis, MO), detecting neurons; rabbit polyclonal antiglial fibrillary acidic protein (GFAP) (DAKO, Carpinteria, CA), labeling astrocytes; and mouse monoclonal anti-RIP (Chemicon, Temecula, CA), marking oligodendrocytes.

The secondary antibodies used were Alexa Fluor 488 chicken anti-rat immunoglobulin G (IgG; Molecular Probes, Inc., Eugene, OR), fluorescein isothiocyanate-conjugated donkey anti-mouse IgG (Jackson ImmunoResearch, West Grove, PA), or Cy3-conjugated donkey anti-rabbit IgG (Jackson ImmunoResearch). Fluorescence was detected using Olympus fluorescence microscopy (BX-52, Olympus, Tokyo, Japan) or with a KEYENCE BZ-8000 (KEYENCE, Osaka, Japan). Omission of primary antibodies served as negative controls, which showed no immunostaining.

### Injection of NPCs

Neurospheres were dispersed in an enzyme solution comprising and resuspended in DMEM/F12 medium to a final concentration of 1.0 × 10^6^ cells/100 μL. The cell suspension (100 μL) was administered via the right femoral vein on day 7 after the cerebral embolism. Vehicle was injected in a similar manner as the NPCs.

### Bromodeoxyuridine labeling

5-Bromo-2′-deoxyuridine (BrdU, Sigma-Aldrich) injection was used in this study to reveal the rate of generation of cells at specific time points after the embolism. In the experiments, rats were administered by the intraperitoneal route a single dose of BrdU (50 mg/kg) at 7 or 28 days after the embolism. Twenty-four hours after the administration, the animals were perfused transcardially with 4% paraformaldehyde under deep anesthesia.

### Histological assessments

On day 7 or 28 after surgery, cerebrally embolized rats were perfused via the heart with 4% paraformaldehyde in 0.1 mol/L phosphate buffer. Their brains were quickly removed and immersed in 30% sucrose in 0.1 mol/L phosphate buffer. The brains were then cut into 5-mm-thick coronal slabs, which were subsequently embedded in Neg50 (Richard-Allan Scientific, Kalamazoo, MI) and cut into 10-μm sections by using a cryostat. For immunostaining, sections were incubated overnight with the desired primary antibody at 4°C after blocking, and then with the corresponding secondary antibody for 1 h. In the case of double immunofluorescence staining, after a wash, the same sections were incubated overnight with another primary antibody at 4°C. Subsequently, they were incubated with the corresponding secondary antibody for 1 h. Omission of primary antibodies served as a negative control. No immunostaining was detected in this group. The following primary antibodies were used: rat monoclonal anti-BrdU (AbD Serotec, Oxford, U.K.), mouse monoclonal anti-rat endothelial cell antigen (RECA) (AbD Serotec, Oxford, U.K.), rabbit polyclonal Ang-1 (Abcam, Minneapolis, MN), rabbit polyclonal anti-GFAP (DAKO, Capinteria, CA), and rat monoclonal anti-GFP (Nacalai Tesque, Kyoto, Japan). The secondary antibodies used were as follow: Alexa Fluor 488 chicken anti-rat IgG (Molecular Probes, Inc., Eugene, OR), Cy3-conjugated goat anti-mouse IgG (Amersham, Buckinghamshire, U.K.), and Cy3-conjugated donkey anti-rabbit IgG (Jackson ImmunoResearch). Fluorescence was detected by using an Olympus fluorescence microscope (BX-52; Olympus) or with a KEYENCE BZ-8000 (KEYENCE).

### Tissue preparation

On day 7 or 28 after surgery, cerebrally embolized or sham rats were sacrificed by decapitation. The whole ipsilateral hemisphere was homogenized in ice-cold buffer containing 320 mmol/L sucrose, 20 mmol/L β-glycerophosphate, 20 mmol/L sodium diphosphate, 0.2 mmol/L sodium orthovanadate, 0.1 mmol/L phenylmethyl sulfonyl fluoride, 5 μg/mL antipain, 5 μg/mL aprotinin, and 5 μg/mL leupeptin at 4°C.

Isolation of brain capillaries was performed by using a modified version of the method described previously (Kago et al. 2006; Takenaga et al. 2009). Rats on day 7 or 28 after the embolism were sacrificed by decapitation, and their whole ipsilateral hemisphere was homogenized in ice-cold 15 mmol/L 2-(4-2[-hydroxyethyl]-1-pioperazinyl)-ethanesulphonic acid (HEPES), pH 7.4, containing 147 mmol/L NaCl, 4 mmol/L KCl, 3 mmol/L CaCl_2_, and 1.2 mmol/L MgCl_2_ (physiological buffer). The homogenate was centrifuged at 3500*g* for 10 min at 4°C, and the resulting pellet was resuspended in physiological buffer containing 20% Ficoll T-400 (Sigma) and then homogenized. After centrifugation at 25,000*g* for 10 min at 4°C, the pellet was resuspended in 15% dextran T-500 (Sigma). The suspension was then layered onto 20% dextran T-500 and centrifuged at 25,000*g* for 10 min at 4°C. The pellet was finally resuspended in physiological buffer and used as the brain capillaries.

### Immunoblotting

Western blotting was performed according to standard protocols. The following primary antibodies were used: rabbit polyclonal antibodies against Ang-1 (Abcam, Minneapolis, MN), Ang-2 (Abcam), Occludin (Life Technologies), ZO-1 (Zymed), Tie2 (Santa Cruz Biotechnology, Inc., Santa Cruz, CA), VEGF (R&D Systems, Inc., McKinley Place, MN), and VEGFR2 (Abcam). Subsequently, the membrane was washed and incubated with secondary antibody. Bound antibody was detected by use of the enhanced chemiluminescence method (Amersham). Quantification was carried out by performing computerized densitometry with an image analyzer (ATTO Co., Tokyo, Japan). To minimize blot variability, we applied an aliquot of pooled “control” homogenate, which was obtained from naïve control rats, to one lane of every gel and calculated the band intensity of immunoblotted samples relative to this standard.

### Statistical analysis

The results were expressed as the means ± standard error of the mean (SEM). Differences between two groups were evaluated statistically by use of the unpaired Student's *t*-test. Statistical comparison among multiple groups was made by performing analysis of variance, followed by Scheffe's test as a post hoc test or repeated-measures analysis of variance. *P*-values of less than 0.05 were considered significant.

## Results

### Characterization of neural progenitor cells

**Figure 1:**
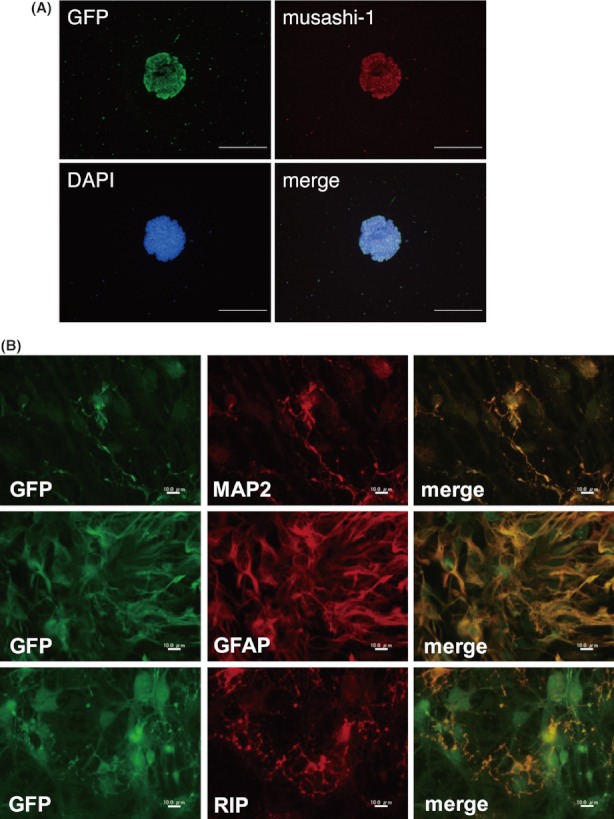
Characterization of neural progenitor cells. (A) Triple staining with green-fluorescent protein (GFP), musashi-1, and 4′,6-diamidino-2-phenylindole (DAPI) was merged and indicated that cells in neurospheres, which were prepared from gestational day 14 fetal rats, expressed the neural progenitor cells markers musashi-1 on day 6 when cultured in vitro. (B) The cells in the neurospheres isolated from GFP transgenic rats differentiated after the withdrawal of growth factors from the medium into MAP2- (neurons), glial fibrillary acidic protein (GFAP)- (astrocytes), and RIP-positive (oligodendrocytes) cells. Scale bars represent 100 μm (A) and 10 μm (B).

Figure 1A shows that cells in the neurospheres expressed the neural progenitor marker musashi-1 on day 6 when cultured in vitro. After triggering in vitro differentiation by withdrawal of the growth factors, we confirmed the tripotent nature of the NPCs by their ability to generate differentiated cells expressing neuronal (MAP2), astrocytic (GFAP), and oligodendrocytic (RIP) markers (Fig. 1B).

### Effect of injection of NPCs on the angiogenesis after ME

**Figure 2:**
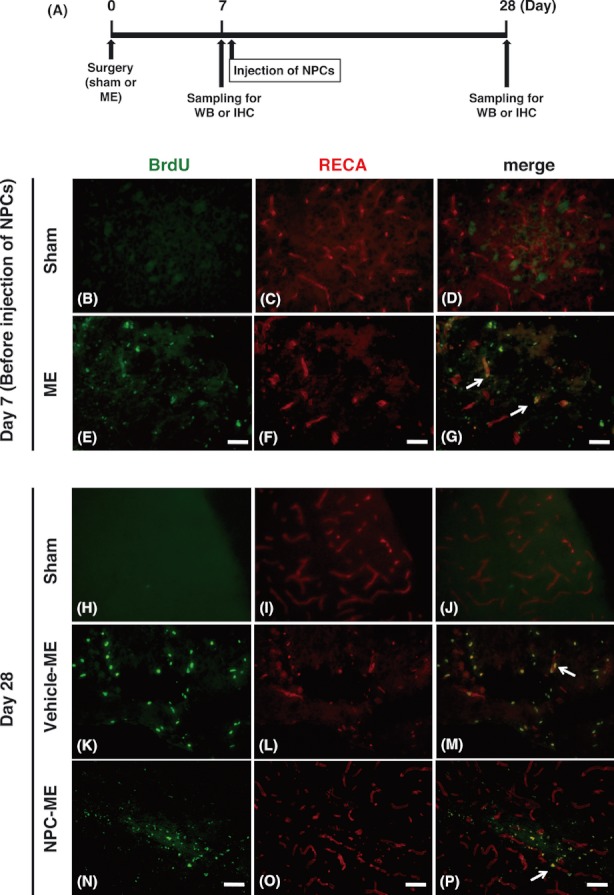
Effects of injection of neural progenitor cells (NPCs) on angiogenesis in the ischemic brain. The experimental protocol in this study is depicted (A). Animals were sacrificed on day 7 or 28 after microsphere embolism-induced cerebral ischemia (ME). Representative images of double staining for bromodeoxyuridine (BrdU; green) and vascular endothelial marker (RECA; red) in the peri-infarct regions of sham-operated (sham; B–D, H–J), ME (E–G) or vehicle-injected ME (Vehicle-ME K–M), and NPC-injected ME (NPC-ME; N–P) rats on days 7 and 28 after ME are shown. White arrows indicate colocalization of BrdU with RECA. Scale bars represent 50 μm.

**Figure 3:**
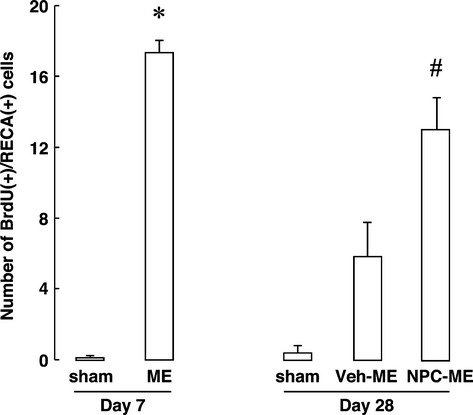
The number of BrdU-positive vascular endothelial cells was counted in the peri-infarct area of sham-operated (sham), microsphere-induced cerebral embolism (ME), vehicle-injected ME (Veh-ME), and neural progenitor cells (NPC) injected ME (NPC-ME) rats on days 7 and 28 after the embolism. All results are presented as the mean ± SEM (*n* = 3–5 rats per group). *Statistically significant difference from sham-operated rats (*P* < 0.05). ^**#**^Statistically significant difference from vehicle-injected ME rats (*P* < 0.05).

To assess the effect of injection of NPCs on the angiogenesis after cerebral ischemia, we measured the number of BrdU-positive vascular endothelial (RECA-positive) cells on days 7 and 28 after the embolism. On day 7 after embolism, a significant increase in the number of BrdU-positive vascular endothelial cells was found in ME rats injected with vehicle (Figs. 2E–G, 3) compared with that of age-matched sham-operated rats (Figs. 2B–D, 3). On the other hand, the number of BrdU-positive vascular endothelial cells in vehicle-injected ME rats on day 28 (Figs. 2M, 3) tended to be decreased compared with that of ME rats on day 7 after the embolism (Figs. 2G, 3). The injection of NPCs attenuated the decrease in the number of BrdU-positive vascular endothelial cells (Figs. 2N–P, 3).

### Effect of injection of NPCs on the levels of VEGF and VEGFR2

**Figure 4:**
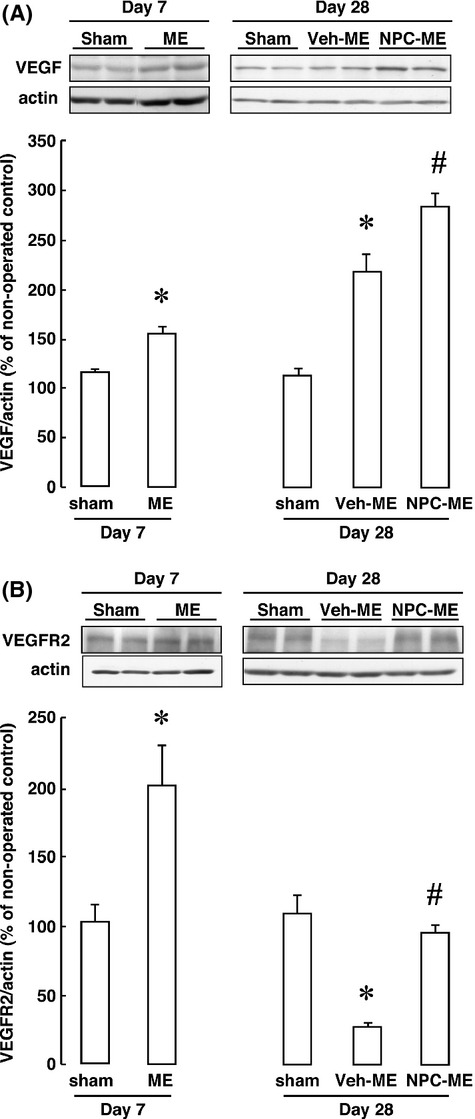
Effect of injection of neural progenitor cells (NPCs) on the levels of vascular endothelial growth factor (VEGF) (A) and its receptor (VEGFR2) (B) proteins after cerebral embolism. Bands corresponding to VEGF and VEGFR2 of sham-operated (sham), microsphere-induced cerebral embolism (ME), vehicle-injected ME (Veh-ME), and NPC-injected ME (NPC-ME) rats on days 7 and 28 after the embolism were scanned; the scanned bands were normalized by actin on the same blot. All results are presented as the mean percentage of the value for the nonoperated control rats ±SEM (*n* = 4–5 rats per group). *Statistically significant difference from sham-operated rats (*P* < 0.05). ^**#**^Statistically significant difference from vehicle-injected ME rats (*P* < 0.05).

Next, we examined the effect of the NPCs on the levels of VEGF and VEGFR2 proteins in the ipsilateral hemisphere of sham-operated and vehicle- or NPC-injected ME rats on days 7 and 28 after surgery. The level of VEGF was increased on day 7 after the embolism compared with that of age-matched sham-operated rats (Fig. 4A). On day 28, the level of VEGF of vehicle-injected ME rats was increased compared with that of age-matched sham-operated rats (Fig. 4a). This increased level was enhanced by NPCs on day 28 after the embolism (Fig. 4A). The level of VEGFR2 was also significantly increased on day 7 and then decreased compared with that of age-matched ME rats on day 28 after the embolism (Fig. 4B). The injection of NPCs attenuated the ischemia-induced decrease in the level of VEGFR2 protein on day 28 after the embolism (Fig. 4B).

### Effect of injection of NPCs on the levels of Ang-1 and Ang-2

**Figure 5:**
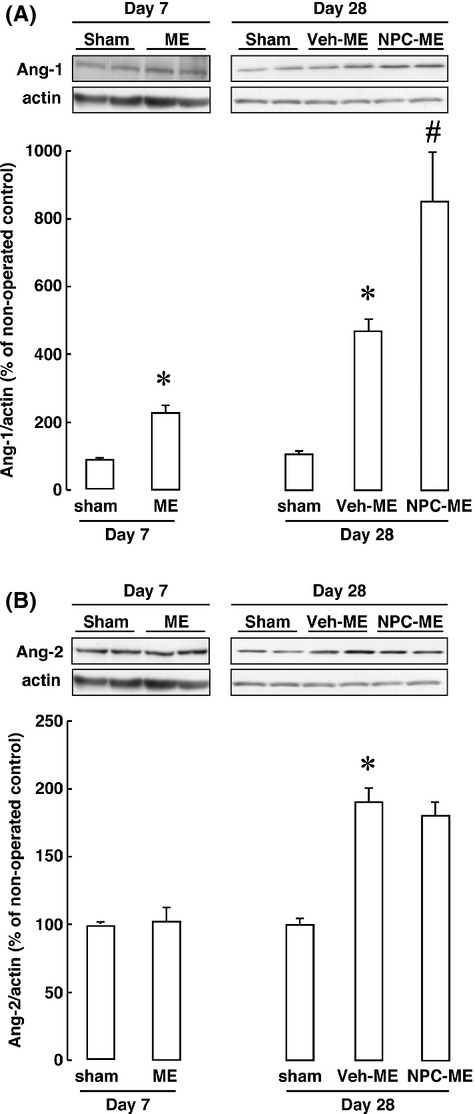
Effect of injection of neural progenitor cells (NPCs) on the levels of angiopoietin-1 (Ang-1) (A) and Ang-2 (B) proteins after cerebral embolism. Bands corresponding to Ang-1 and Ang-2 of sham-operated (sham), microsphere-induced cerebral embolism (ME), vehicle-injected ME (Veh-ME), and NPC-injected ME (NPC-ME) rats on days 7 and 28 after the embolism were scanned; the scanned bands were normalized by actin on the same blot. All results are presented as the mean percentage of the value for the nonoperated control rats ±SEM (*n* = 4–5 rats per group). *Statistically significant difference from sham-operated rats (*P* < 0.05). ^**#**^Statistically significant difference from vehicle-injected ME rats (*P* < 0.05).

We next examined the effect of the NPCs on the levels of Ang-1, Ang-2, and Tie2 proteins in the ipsilateral hemisphere of sham-operated and vehicle- or NPC-injected rats on days 7 and 28 after surgery. The level of Ang-1 proteins in ME rats on day 7 was significantly increased compared with that of age-matched sham-operated rats (Fig. 5A). On day 28 after the embolism, Ang-1 level was still increased compared with that in age-matched sham-operated rats (Fig. 5A). The injection of NPCs led to a further increase in the level of Ang-1 protein compared with that in vehicle-injected ME rats on day 28 (Fig. 5A). In contrast, there were no significant differences in the level of Ang-2 proteins between sham-operated and ME rats on day 7 (Fig. 5B). Although the level of Ang-2 protein of vehicle-injected ME rats was increased compared with that of age-matched sham-operated rats on day 28, there were no significant differences in the level of Ang-2 protein between vehicle-injected and NPC-injected ME rats on day 28 (Fig. 5B).

### Effect of injection of NPCs on the levels of Tie2 in brain capillaries

**Figure 6:**
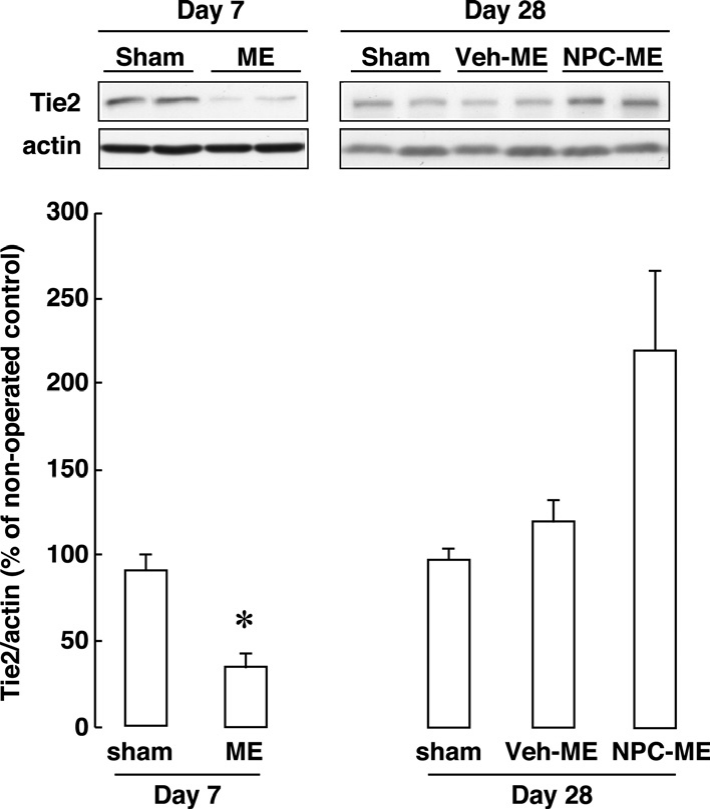
Effect of injection of neural progenitor cells (NPCs) on the level of Tie2 protein in brain capillaries after cerebral embolism. Bands corresponding to Tie2 of sham-operated (sham), microsphere-induced cerebral embolism (ME), vehicle-injected ME (Veh-ME), and NPC-injected ME (NPC-ME) rats on days 7 and 28 after the embolism were scanned; the scanned bands were normalized by actin on the same blot. All results are presented as the mean percentage of the value for the nonoperated control rats ±SEM (*n* = 4 rats per group). *Statistically significant difference from sham-operated rats (*P* < 0.05).

The Ang-1/2 receptor Tie2 is expressed predominantly in vascular endothelial cells. Therefore, we examined the effect of the NPCs on the levels of Tie2 proteins in brain capillaries of sham-operated and vehicle- or NPC-injected rats on days 7 and 28 after surgery. The level of Tie2 proteins was decreased on day 7 after the embolism but returned to the level of sham-operated rats on day 28 (Fig. 6). NPC injection tended to increase the level of Tie2 protein compared with that of vehicle-injected ME rats on day 28 (Fig. 6).

### Effect of injection of NPCs on the levels of occludin and ZO-1 in brain capillaries

**Figure 7:**
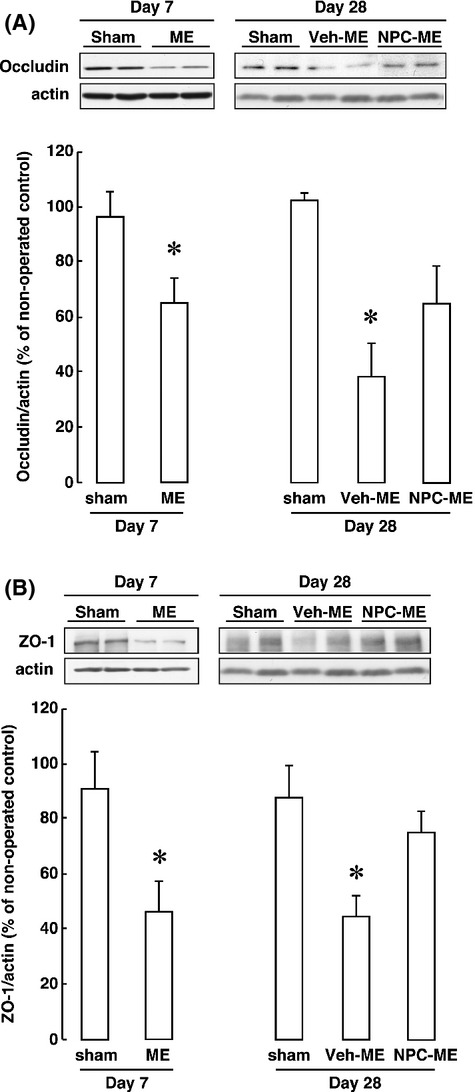
Effect of injection of neural progenitor cells (NPCs) on the levels of occludin (A) and ZO-1 (B) proteins in brain capillaries after cerebral embolism. Bands corresponding to occludin and ZO-1 of sham-operated (sham), microsphere-induced cerebral embolism (ME), vehicle-injected ME (Veh-ME), and NPC-injected ME (NPC-ME) rats on days 7 and 28 after the embolism were scanned; the scanned bands were normalized by actin on the same blot. All results are presented as the mean percentage of the value obtained for the nonoperated control rats ±SEM (*n* = 4 rats per group). *Statistically significant difference from sham-operated rats (*P* < 0.05).

We further examined the level of the tight junctional proteins occludin and ZO-1, which are found in the brain capillaries of the blood–brain barrier. The level of occludin was decreased compared with that in age-matched sham-operated rats on days 7 and 28 after surgery (Fig. 7A). Injection of NPCs tended to attenuate the decrease in the level of occludin on day 28 after the embolism, although there was no significant difference (Fig. 7A). The level of ZO-1 was also decreased on day 7 after the embolism and remained at that value up to day 28 (Fig. 7B). The injection of NPCs tended to inhibit the reduction in the amount of ZO-1 protein in brain capillaries on day 28 after the embolism (Fig. 7B).

### Effect of injection of NPCs on the localization of Ang-1 and phenotype of injected NPCs

**Figure 8:**
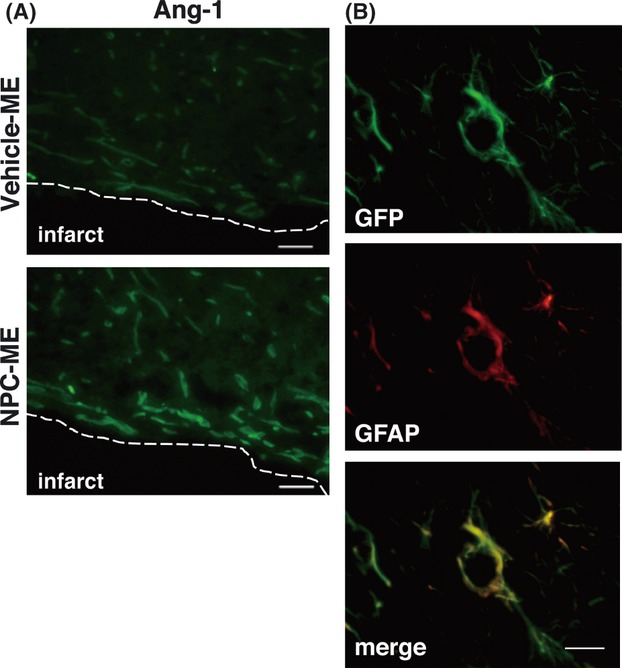
Histological analysis of neural progenitor cell (NPC)-injected brain after the embolism. Effect of injection of NPCs on the expression of angiopoietin-1 (Ang-1) and glial fibrillary acidic protein (GFAP) after cerebral embolism. Ang-1 protein is expressed in the peri-infarcted areas of vehicle-injected microsphere-induced cerebral embolism (Veh-ME) and NPC-injected ME (NPC-ME) rats on day 28 after the embolism (A). The green-fluorescent protein (GFP)-positive injected NPCs express astrocyte marker GFAP on day 28 after the embolism (B). Scale bars represent 50 μm (A) and 20 μm (B).

The expression of Ang-1 in vehicle- and NPC-injected ME rats on day 28 after the embolism was examined histologically. As shown in Figure 8A, the expression of Ang-1 protein in the peri-infarcted area was increased by the injection of NPCs. Furthermore, a few injected NPCs, which were identified as GFP-positive cells, expressed GFAP in the peri-infarcted areas (Fig. 8B), although most injected NPCs were still positive for the NPC marker musashi-1 on day 28 after the embolism (not shown).

## Discussion

In this study, we showed an increase in the number of BrdU-positive vascular endothelial cells in the peri-infarct region on day 7 after cerebral embolism. Although the number of these cells tended to be increased compared with that for the sham-operated rats on day 28, the ability of the endothelial cells to proliferate was attenuated compared with that at day 7. Taken together, our data indicate that long-term and severe cerebral embolism enhanced endogenous angiogenesis transiently and then suppressed it at later stages. In this study, the intravenous injection of NPCs promoted angiogenesis in the peri-infarct area even on day 28 after the embolism, which increase was accompanied by the alteration of angiogenic factors and their receptors. Although angiogenesis was required for protection of infarct area in the ischemic brain (Richardson et al. 2001), our previous results demonstrated that the injection of NPCs does not repair the injured tissue after an embolism (Moriyama et al. 2011). Therefore, NPC-induced angiogenesis at the later stage may contribute to improvement of ischemia-induced brain dysfunction rather than have a restorative effect on the infarcted areas.

As VEGF and Ang play a pivotal role in the angiogenesis, we further investigated changes in the level of these angiogenic factors and their receptors. In this study, the levels of VEGF and VEGFR2 were increased on day 7 after the embolism. It has been suggested that angiogenesis in ischemic tissues is promoted by VEGF, the expression of which is upregulated by hypoxia-inducible factor 1α (HIFα), via VEGFR2 signaling (Marti et al. 2000). In this sense, the expression of HIFα might have been increased in our vehicle-injected ME rats, as ME is reported to induce a sustained decrease in the cerebral blood flow in the ipsilateral hemisphere (Miyake et al. 1993). Therefore, microsphere embolism-induced angiogenesis on day 7 might have been due to the increased level of VEGF proteins in response to the ischemic condition, although the underlying mechanism for the increased level of VEGFR2 on day 7 remains to be determined. We also demonstrated that the injection of NPCs further increased the level of VEGF compared with that of vehicle-injected ME rats on day 28. In addition, the level of VEGFR2 was maintained at that of the age-matched sham-operated rats by injection of NPCs. Thus, long-term angiogenic activity after cerebral embolism might be mediated by the development and maintenance of VEGF/VEGFR2 signaling. Although the source of the VEGF increased by NPCs remains to be determined, it has been found that treatment of astrocytes with bone marrow-derived stromal cells stimulates the expression of VEGF in the astrocytes (Zacharek et al. 2007). In this regard, it is interesting to note that GFP-positive cells, which were the NPCs injected, had differentiated into GFAP-positive cells in the peri-infarcted areas by day 28.

In this study, the level of Ang-1 after the embolism was time-dependently increased. In addition, the expression of Ang-1 was enhanced by injection of NPCs, whereas the level of Ang-2 was unchanged by NPCs compared with that of vehicle-injected ME rats on day 28. Ang-1 function is an agonist for Tie2, whereas Ang-2 is antagonistic toward Tie2. Therefore, the shift of the balance between Ang-1 and Ang-2 has been implicated in enhanced angiogenic activity after an embolism. As Tie2 is expressed predominantly in endothelial cells, we further examined the level of Tie2 protein in the brain capillaries isolated from microsphere-embolized rats. The NPC-induced increase in Tie2 proteins in brain capillaries on day 28 may have contributed to the ability of NPCs to enhance blood vessel formation and maintenance for a long period after the embolism. Ang-1 induces the gene expression of occludin, which is one of the tight junctional proteins in brain capillary endothelial cells, by acting through Tie2 (Lee et al. 2003; Hori et al. 2004). It is noteworthy that the injection of recombinant human Ang-1 increases the expression of occludin and ZO-1 in the ischemic brain (Yu et al. 2012). Therefore, the relatively high level of Ang-1 on day 28 after the NPC injection compared with that of Ang-2 might have promoted the expression of occludin and ZO-1 in brain capillary endothelial cells, which proteins have been implicated in vessel formation and vascular stabilization. This interpretation is consistent with the finding that the injection of NPCs tended to increase the amount of ZO-1 protein in brain capillaries on day 28 after the embolism.

In conclusion, as marked expression of Ang-1 after injection of NPCs occurred in the peri-infarct area on day 28, intravenous injection of NPCs had the ability to promote angiogenesis for a long period through Ang-1/Tie2 and/or VEGF/VEGFR2 signaling even when the injection was started on day 7 after the embolism. These NPC-induced angiogenic activities may be involved in vessel stabilization and maintenance as well as in vessel formation for a long period after the embolism. They may be associated with the improvement of brain dysfunction after cerebral embolism, including learning and memory dysfunction and depression-like behavior.
